# Boosting Piezocatalytic Performance of BaTiO_3_ by Tuning Defects at Room Temperature

**DOI:** 10.3390/nano14030276

**Published:** 2024-01-29

**Authors:** Donghui An, Renhong Liang, Hua Liu, Chao Zhou, Mao Ye, Renkui Zheng, Han Li, Shanming Ke

**Affiliations:** 1 School of Physics and Materials Science, Guangzhou University, Guangzhou 510006, China; 355700210015@email.ncu.edu.cn (D.A.); nculrh@163.com (R.L.); lh245711@e.gzhu.edu.cn (H.L.); 2112319083@e.gzhu.edu.cn (C.Z.); yem001@gzhu.edu.cn (M.Y.); zrk@gzhu.edu.cn (R.Z.); 2 School of Physics and Materials Science, Nanchang University, Nanchang 330031, China

**Keywords:** piezocatalysis, oxygen vacancies, room temperature lithium reduction, ultrasonic cavitation, multi-filed catalysis

## Abstract

Defect engineering constitutes a widely-employed method of adjusting the electronic structure and properties of oxide materials. However, controlling defects at room temperature remains a significant challenge due to the considerable thermal stability of oxide materials. In this work, a facile room-temperature lithium reduction strategy is utilized to implant oxide defects into perovskite BaTiO_3_ (BTO) nanoparticles to enhance piezocatalytic properties. As a potential application, the piezocatalytic performance of defective BTO is examined. The reaction rate constant increases up to 0.1721 min^−1^, representing an approximate fourfold enhancement over pristine BTO. The effect of oxygen vacancies on piezocatalytic performance is discussed in detail. This work gives us a deeper understanding of vibration catalysis and provides a promising strategy for designing efficient multi-field catalytic systems in the future.

## 1. Introduction

With the development of industrialization and urbanization, the pollution of water resources by organic pollutants has caused severe environmental problems. This issue even threatens human health and life [1,2]. Therefore, it is imperative to address wastewater treatment and enhance the ecological environment. Recently, piezocatalysis has gained significant attention due to its ability to degrade organic pollutants and purify water through the abundant mechanical vibration in nature [3,4,5,6,7]. During a typical piezocatalysis process, electron and hole pairs are generated on the surface of the piezoelectric material under mechanical stress and separated by the piezoelectric potential. Subsequently, a series of oxidation/reduction reactions occur to produce reactive radicals to decompose the dye molecules [8,9]. Many piezoelectric materials, such as BaTiO_3_ [10,11,12,13], ZnO [14], KNbO_3_ [15], BaSrTiO_3_ [16], and ZnSnO_3_ [17], have been extensively investigated as efficient catalysts for the degradation of organic pollutants and water splitting. However, compared with the catalytic performance of electrocatalysis and photocatalysis, the catalytic activity of these piezoelectric materials is currently unsatisfactory, which limits their practical applications [18,19]. Therefore, there is an urgent need to develop novel strategies to enhance their catalytic activity [20,21,22,23]. As the piezoelectric effect solely contributes to the charge separation rather than the excitation of electron–hole pairs, the carrier concentration plays a crucial role in enhancing piezocatalytic performance [3,4,5,6,7,8,9]. Oxygen vacancies are commonly occurring defects in oxides and can be utilized to modulate the carrier concentrations [24,25]. Recently, Wang et al. [17] reported a hydrogen reduction approach used to generate surface defects in ZnSnO_3_ nanowires, which demonstrated remarkable piezocatalytic activity through capitalizing on well-modulated oxygen vacancies. Barium titanate nanoribbons with defects exhibit improved piezocatalytic performance owing to their increased carrier concentrations derived from oxygen vacancies [26]. These findings suggest that the modulation of oxygen vacancies represents a viable and crucial approach for improving the piezocatalytic activity of piezoelectric oxides [27,28].

So far, the generation of oxygen vacancies in piezocatalysts has mainly been achieved through heating treatment under vacuum [26,29] or hydrogen atmosphere [30,31]. However, these methods have limitations, such as harsh reaction conditions (e.g., high temperature and pressure) and complex synthesis procedures. Therefore, there is a need for an efficient and scalable synthesis method for piezocatalysts with controlled oxygen concentration at room temperature, along with the optimization of their piezocatalytic activities for practical applications. In this regard, Ou and co-workers proposed a simple approach for the synthesis of black TiO_2_ using a lithium reduction method [32]. The lithium reduction strategy offers several advantages, including all-room-temperature processing, controllability, time efficiency, versatility, and scalability. It is reasonable to expect that the lithium reduction method could also be an effective approach for modulating oxygen vacancies in piezoelectric oxides.

In this study, the effect of lithium reduction on the piezocatalytic performance of BaTiO_3_ (BTO) nanoparticles was investigated. BTO nanoparticles with varying concentrations of oxygen vacancy were obtained by grinding raw powders with different proportions of lithium powders at room temperature. The oxygen vacancies were characterized using scanning transmission electron microscopy, and the piezoelectric response of the samples was demonstrated by piezoresponse force microscopy. The optimized piezocatalysts exhibited a degradation efficiency of up to 97% within a 20 min timeframe. We propose a mechanism for ultrasonic vibration-driven piezocatalysis and provide a comprehensive understanding of the influence of oxygen vacancies on the synergistic coupling effect of piezocatalysis and ultrasonic cavitation.

## 2. Materials and Methods

### 2.1. Preparation of Catalysts

The used raw materials are commercially available Barium titanate nanopowders (BaTiO_3_, 99.9% metals basis, <100 nm), passivated Li powders (99.9%), and dimethyl carbonate (DMC, 99.95%). All reagents were directly used without further purification.

In this study, 5 g of Barium titanate (BTO) and a specific proportion of passivated lithium powder were weighed and placed into a mortar for grinding. To ensure thorough grinding, an appropriate amount of dimethyl carbonate (DMC) was added as a dispersant. The grinding process was conducted in a glove box filled with Ar gas to prevent oxidation of the lithium powder by air. After grinding for one hour, the sample was removed from the mortar and washed with dilute hydrochloric acid (HCl) to dissolve the lithium oxide generated during the reduction process. The sample was then subjected to centrifugation and washed three times with deionized water. The resulting sample was denoted as BTO-x (x wt% = 0 wt%, 1 wt%, 3 wt%, 5 wt%, 7 wt%, and 10 wt%, where x represents the weight percentage (wt%) of lithium powder used in the reduction process). By adjusting the ratio of lithium metal powder, the defect content in the BTO nanoparticles was controlled. The reduction process occurred spontaneously at room temperature, as indicated in Equation (1).

(1) $$BaTiO_{3}+2xLi\to BaTiO_{3-x}+x{Li}_{2}O$$

### 2.2. Characterization

An X-ray diffractometer (SmartLab, Rigaku, Tokyo, Japan) was used to investigate the phase structure of the BaTiO_3_ samples. The absorbance and diffuse reflectance properties were examined using a UV-Vis spectrophotometer (UH4150, HITACHI, Tokyo, Japan). Raman spectra were collected at room temperature using a confocal microscope spectrometer (WITec Alpha300 Raman, Ulm, Germany) with a 532 nm laser source. A probe aberration-corrected STEM microscope [FEI Titan 80–300 (Hillsboro, OR, USA) operating at 300 kV and JEOL JEM-ARM200CF (Tokyo, Japan) operating at 200 kV] was utilized. X-ray photoelectron spectroscopy (XPS) was performed using a Thermo Fischer (Waltham, MA, USA), ESCALAB Xi+ instrument. High-resolution transmission electron microscopy (HRTEM), energy-dispersive X-ray spectroscopy (EDS), STEM high-angle annular dark-field (HAADF) observation, and electron energy loss spectroscopy (EELS) were conducted on an FEI Titan 80–300 microscope to probe the structural and chemical evolution of samples. Atomic force microscopy (Cypher ES, Oxford, Santa Barbara, CA, USA) coupled with piezoelectric reaction force microscopy (PFM) was employed to perform piezoelectric reaction measurements on the prepared samples. Photoluminescence (PL) measurements were carried out using an FLS900 fluorimeter (Edinburgh Instruments, Livingston, UK). Electrochemical impedance spectra (EIS) were obtained using the CHI660E electrochemical analyzer (Chenhua, Shanghai, China). Electron paramagnetic resonance (EPR) spectra were recorded by a Bruker A300 (Freiberg, Germany) paramagnetic resonance spectrometer at room temperature.

### 2.3. Piezocatalytic Activity Experiments

The piezocatalytic activities of the BTO-x samples were evaluated by decomposing Rhodamine B (RhB) with an ultrasonic cleaner (150 W and 40 kHz). Representatively, 0.05 g of the BTO-x powders was dispersed into 50 mL of RhB solution (5 mg/L). The mixed solution was stirred in the dark for 30 min to allow for adsorption and desorption equilibrium before ultrasonic irradiation to balance adsorption-desorption. Then, a 50 mL glass beaker containing the mixed suspension was fixed in a specific position within an ultrasonic cleaner. To monitor the degradation process, 3 mL of the clarified solution was sampled every 5 min and subjected to centrifugation to segregate the BTO-x powders. The concentration of the dye in the aqueous solution was determined using a UV-Vis spectrophotometer. The regularity of the degradation process was confirmed by repeating the experiments under the same conditions. In addition, the synergistic catalytic activity of barium titanate samples under ultraviolet (UV) was evaluated using an ultraviolet (125 W, 365 nm) light source. Furthermore, the degradation experiments were repeated using recovered BTO-5 to evaluate the cycling stability of the piezoelectric catalyst for RhB degradation.

## 3. Results

**Figure 1:**
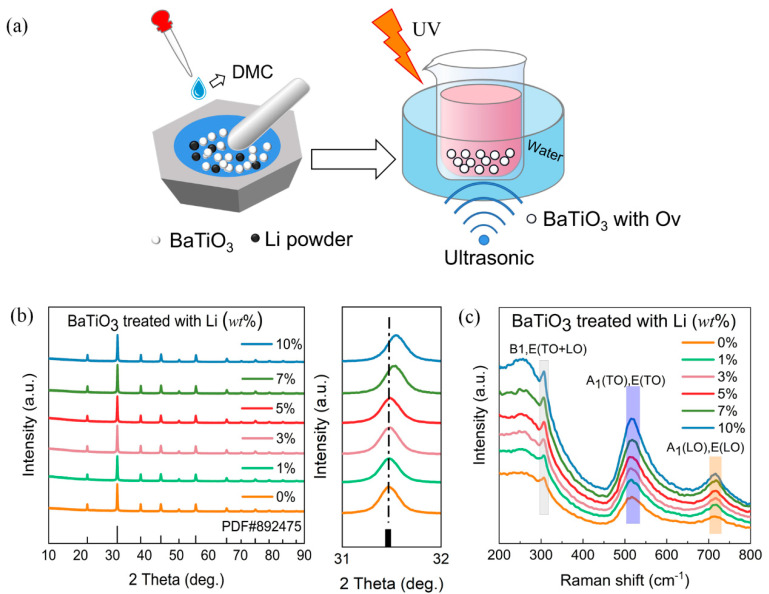
(**a**) The schematic of lithium reduction treatment and piezocatalytic test. (**b**) XRD patterns and the selected diffraction peak near 31.5° of the BTO-x samples. (**c**) Room-temperature Raman spectra of the BTO-x samples.

Figure 1a provides a schematic representation of the lithium reduction treatment and piezocatalytic processes of the BaTiO_3_ nanoparticles. The X-ray diffraction (XRD) patterns of the BTO-x samples, as shown in Figure 1b, exhibit a good match with pseudo-cubic BTO (PDF#89-2475). The diffraction peaks of all samples demonstrate the high crystallinity and purity of BTO, without any impurity such as Li_2_O. This indicates that the intrinsic crystal structure of the barium titanate nanoparticles remains unchanged after the lithium reduction process. However, there is a gradual shift of the main diffraction peak to a higher angle with increasing Li content, indicating the implantation of oxygen vacancies into the BTO nanoparticles and an expansion of the unit cell. The expansion of the lattice due to the presence of oxygen vacancies is a well-known phenomenon observed in oxide materials. Oxygen vacancies typically cause an overall expansion of the oxide lattice on both short- and long-range scales [33,34]. The room-temperature Raman vibrational spectroscopy experiments were conducted to examine the local distortions of the lattice and confirm the crystal structures. Figure 1c shows the Raman spectra of all the analyzed samples. The characteristic peaks centered around 306, 516 and 716 cm^−1^ could be assigned to the B1, E(TO+LO); E, A1(TO); and E, A1(LO) modes, respectively. These peaks indicate the presence of an acentric structure resulting from structural disorder of the Ti^4+^ ions within the [TiO_6_] octahedra [35,36,37,38]. It is well known that the ideal cubic BTO is Raman inactive due to the isotropic distribution of electrostatic forces around the Ti^4+^ ions within each octahedron [39]. However, the presence of tetrahedral distortion of [TiO_6_] octahedra can induce piezoelectric effects, even though the X-ray diffraction patterns of BTO suggest a global cubic-like symmetry [40,41,42].

**Figure 2:**
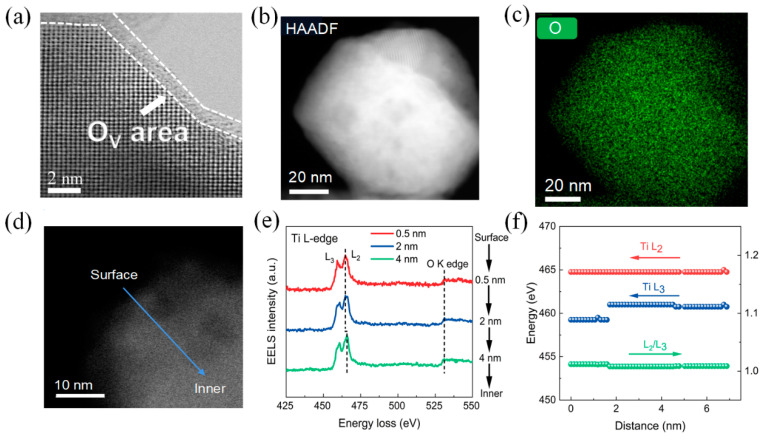
(**a**) BF-STEM image of a particle from the BTO-5 sample. (**b**) HAADF-STEM image of a particle from the BTO-5 sample. (**c**) the EDS elemental maps of O. (**d**) HAADF-STEM image of a particle from the BTO-5 sample with the EELS line-scan. (**e**) EELS spectra of the Ti-*L*_2,3_ and O-K edges taken along the blue line in (**d**), where the black dotted lines show the main peaks of the Ti-*L*_2,3_ and O-K edges. (**f**) Ti-*L*_3_ and Ti-*L*_2_ energy profiles and *L*_2_/*L*_3_ intensity ratio as a function of position.

In Figure S1, scanning electron microscope (SEM) images revel that the particle size of the BTO samples remains almost the same before and after the lithium reduction treatment. Additionally, the surface morphology of BTO is not altered after grinding with lithium powders. To further examine the detailed morphology and microstructure of BTO-5, aberration-corrected scanning transmission electron microscopy (STEM) was employed. As shown in Figure 2a, BTO-5 displays a crystalline-disordered core-shell structure. A distinct disordered layer with a thickness of approximately 1 nm is clearly visible in the STEM image. The elemental map of oxygen obtained through energy-dispersive X-ray spectroscopy (EDS) (Figure 2b,c) reveals that the disordered layer has a lower oxygen content compared to the interior of the BTO particle. This indicates that oxygen vacancies have been introduced into the surface of the BTO as a result of the reduction of surface lattice oxygen by lithium at room temperature.

To further investigate the presence of oxygen vacancies in the surface layer of the BTO-5 sample, atomic-resolution electron energy loss spectroscopy (EELS) line-scanning was conducted from the surface to the bulk of the particle. Figure 2d displays a high-angle annular dark-field scanning transmission electron microscopy (HAADF-STEM) image with the EELS line-scan range indicated for a BTO-5 particle. Representative EELS spectra of the Ti-*L*_2,3_ and O-K edges in three different regions, spanning from the surface to the bulk, are highlighted in Figure 2e. All spectra are normalized to the Ti *L*_2_ peak. The Ti-*L*_3_ and Ti-*L*_2_ peaks exhibit a noticeable energy shift towards higher energy at the surface compared to the core region (Figure 2e,f). In addition, the intensity ratio of *L*_2_/*L*_3_ decreases from the surface to the inner part, indicating that the Ti oxidation state at the surface is lower than in the inner region. This suggests a reduction in the Ti4+ ions at the surface layer. Furthermore, the intensity of the O-K edge peak is suppressed, indicating a decrease in the oxygen content at the surface. This suppression of the O-K edge peak is attributed to the formation of oxygen vacancies in the surface layer [43,44,45,46,47].

**Figure 3:**
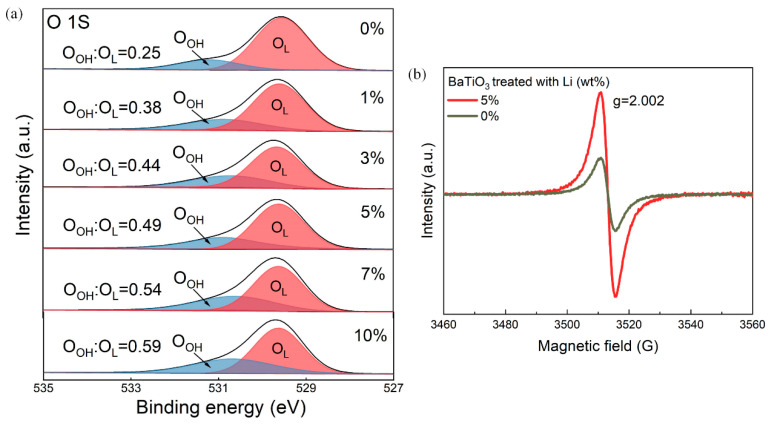
(**a**) O 1s XPS spectra of BTO-x samples. (**b**) EPR spectra of selected BTO-x samples.

The presence of oxygen vacancies can be further confirmed through X-ray photoelectron spectroscopy (XPS) and electron paramagnetic resonance spectroscopy (EPR) studies. Figure 3a shows the O 1s XPS spectra of the BTO-x samples. The O 2p peak at ~529.7 eV is attributed to the lattice oxygen, while the peak at ~530.8 eV corresponds to the signal of surface hydroxyl groups, indicating the presence of oxygen vacancies [48,49]. The percentage of O 1s peaks assigned to hydroxyl groups gradually increases from BTO-0 to BTO-10, indicating an increase in the concentration of oxygen vacancies. It is worth noting that the lithium-reduced BTO samples do not contain any detectable lithium element, as confirmed by the absence of a Li 1s spectrum (Figure S2). This suggests that the lithium oxide was completely removed during the acid treatment. Furthermore, the EPR signal of oxygen vacancy is clearly observed at g = 2.002 in the Li-treated BTO samples, indicating the presence of oxygen vacancies (Figure 3b).

**Figure 4:**
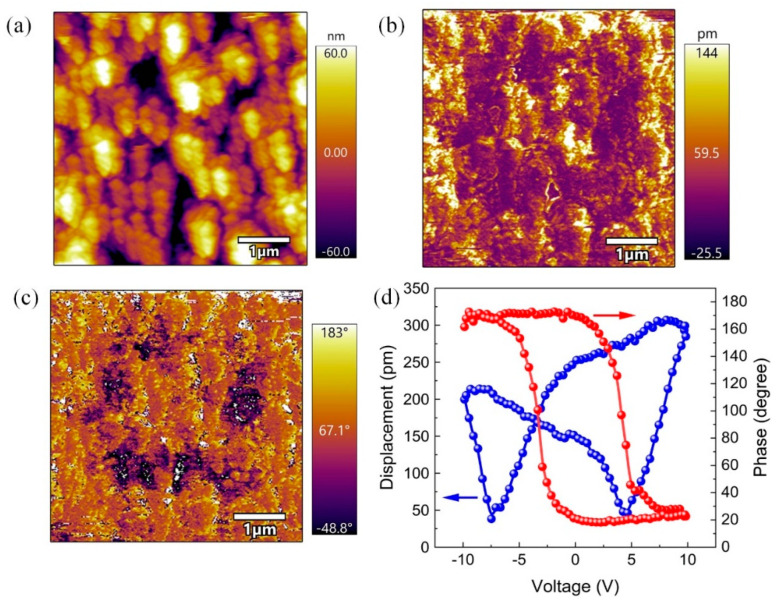
PFM results of BTO-5. (**a**) Topography image, (**b**) amplitude image, (**c**) phase image, and (**d**) the electric displacement and phase hysteresis loops.

The piezoelectric properties of BTO nanoparticles were characterized and confirmed using piezoresponse force microscopy (PFM). Figure 4 presents the topography image and piezoelectric response of the BTO-5 sample. A typical ferroelectric butterfly amplitude curve is observed, along with a well-defined 180° phase change hysteresis loop. These observations provide clear evidence of the piezoelectricity of the BTO-5 sample.

**Figure 5:**
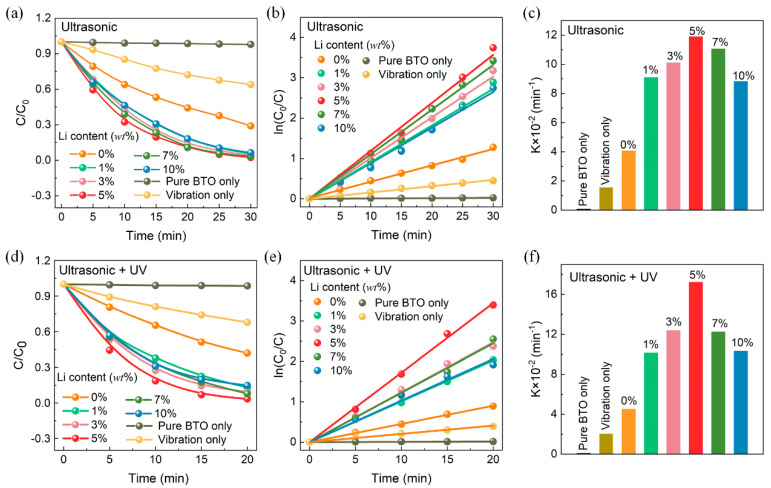
Effect of Li-treated BTO on the degradation of RhB. (**a**) The relative concentration ratios C/C_0_ of RhB with different piezocatalysts and vibration state in the dark. (**b**) Plots of ln(C_0_/C) versus the ultrasonic vibration time in the dark. (**c**) The corresponding reaction kinetics rate constant k values according to (**b**). (**d**) The C/C_0_ of RhB under ultrasonic vibration and UV irradiation simultaneously. (**e**) Plots of ln(C_0_/C) versus the ultrasonic vibration time under ultrasonic vibration and UV irradiation, and (**f**) the corresponding reaction kinetics rate constant k.

The piezocatalytic activities of the BTO-x samples were evaluated by degrading Rhodamine B (RhB) dye under ultrasonic vibration at a frequency of 40 kHz and a power of 150 W under dark conditions, as shown in Figure 5a–c. According to the first-order kinetics, the degradation efficiency rate constant *k* is obtained from the slope of the ln(C_0_/C)-time diagram. In the absence of any vibration, there was negligible dye degradation, indicating the essential role of vibration in the process. When ultrasonic vibration was applied without any catalyst, RhB degradation occurred to some extent, which can be attributed to the ultrasonic cavitation effect [50]. For the BTO-x samples, the piezocatalytic activity exhibited a trend of initially increasing and then decreasing, with the peak observed at 5 wt% Li-treated BTO. BTO-5 demonstrated superior piezocatalytic activity, with a *k* value of 0.1189 min^−1^. It is worth noting that defects have multiple effects on piezocatalytic properties. By increasing the amount of lithium powder during the room temperature process, more oxygen vacancies can be created, resulting in a higher concentration of carriers on the BTO surface. The piezocatalytic performance is generally proportional to the defect content, as it enhances carrier concentration, oxygen adsorption, and active sites. However, excess defects can be detrimental to piezocatalytic activity. They can act as charge recombination sites and reduce the piezoelectric polarization [51,52,53], thus negatively impacting the overall performance. Therefore, there is an optimal defect concentration that maximizes the piezocatalytic activity, as observed in the peak performance of the 5 wt% Li-treated BTO sample.

The influence of UV illumination alone on the experiments was explored, as shown in Figure S3. The results indicate that the photocatalytic performance of BTO nanoparticles is mediocre due to their large band gap (3.4 eV), regardless of the presence of oxygen vacancies. The absorption spectroscopy results in Figure S4 demonstrate that the band gap of BTO barely changes before or after Li treatment. The catalytic performance of BTO under the combined effect of UV irradiation and ultrasonic vibration is shown in Figure 5d–f. It was observed that the concentration of RhB decreased rapidly under the combination of UV irradiation and ultrasonic vibration. Among the BTO-x piezocatalysts, BTO-5 exhibited the highest reaction rate constant of 0.1721 min^−1^, which is nearly four times that of pristine BTO. The *k* value is superior to that observed in other BTO-based experiments, listed in Table 1. Furthermore, the BTO-x piezocatalysts demonstrated favorable cyclic stability in terms of crystal structure, morphology, and piezocatalysis, as shown in Figure S5.

**Figure 6:**
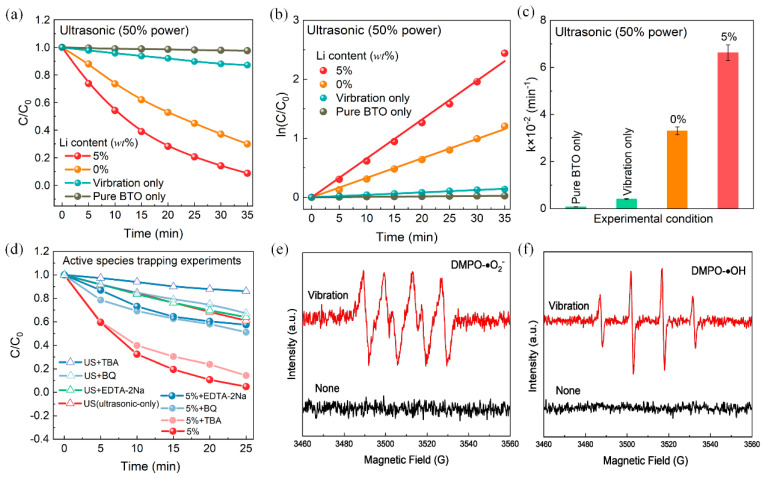
(**a**–**c**) Catalytic performance of BTO under ultrasonic vibration with a power of 75 W. (**d**) Active species trapping degradation experiments for catalysis system. (**e**) The ESR spectra of DMPO-·O^2−^ and (**f**) DMPO-·OH using BTO-5 piezocatalyst.

The effect of ultrasonic power on the catalytic performance was further investigated, taking into account the ultrasonic cavitation effect. Figure 6a–c illustrate the degradation of RhB under ultrasonic vibration (40 kHz, 75 W) in the dark. When the ultrasonic power was set at 150 W, the degradation rate of RhB by BTO-5 reached 98% within 30 min. However, when the ultrasonic power was halved to 75 W, the degradation rate decreased to 86% within the same time period. The reduction in ultrasonic energy not only weakens the ultrasonic cavitation effect but also reduces the stress and weakens the piezocatalytic effect. This leads to a suppression in the degradation efficiency of RhB.

## 4. Discussion

To investigate the role of active species in the ultrasonic vibration degradation of dyes, different scavengers (0.05 mmol) were used. Figure 6d compares the degradation efficiency with and without scavengers under ultrasonic vibration-only conditions. The addition of tert-butanol (TBA), an ·OH scavenger, significantly reduced the degradation efficiency. This suggests that ·OH radicals are the primary active species involved in the degradation of RhB molecules caused by the ultrasonic cavitation effect. It is well-known that ·OH radicals can be generated from the dissociation of water molecules during the cavitation process induced by ultrasound [57]. On the other hand, the addition of benzoquinone (BQ, an ·O^2−^ scavenger) and ethylenediaminetetraacetate disodium (EDTA-2Na, a h^+^ scavenger) only slightly decreased the degradation efficiency. This indicates that ·O^2−^ and h^+^ radicals play a secondary role in the overall piezocatalytic degradation of RhB molecules. When BQ and EDTA-2Na were separately added to the piezocatalysis system, the degradation efficiency was significantly reduced, resulting in a degradation rate of only 50% for RhB within 25 min. This inhibitory effect can be attributed to the scavenging of ·O^2−^ and h^+^ radicals, demonstrating that these species are the main active substances in the piezocatalytic degradation of RhB molecules. In addition, the active species were confirmed via electron spin resonance (ESR). Figure 6e,f show the ESR spectra of DMPO-·O^2−^ and DMPO-·OH, respectively. The DMPO-·O^2−^ spectra exhibited four groups of strong signals generated by ultrasonic vibrations, confirming the generation of ·O^2−^ active species during the degradation process. Similarly, the ESR spectra of DMPO-·OH indicated the generation of ·OH radicals. In conclusion, the results confirm that both ·O^2−^ and ·OH reactive species are generated during the degradation process. During the piezocatalytic process, the following chemical reactions are believed to occur:

(2) $$H_{2}O\overset{vibration}{\to }\cdot H+\cdot OH$$

(3) $$O_{2}\overset{vibration}{\to }2\cdot O,$$

(4) $$O_{2}+H_{2}O\to 2\cdot OH,$$

(5) $$BTO\overset{vibration}{\to }BTO(e^{-}+h^{+}),$$

(6) $$O_{2}+e^{-}\to \cdot O_{2}^{-},$$

(7) $$RhB+\cdot \frac{O_{2}^{-}}{h^{+}\to degradation}$$

**Figure 7:**
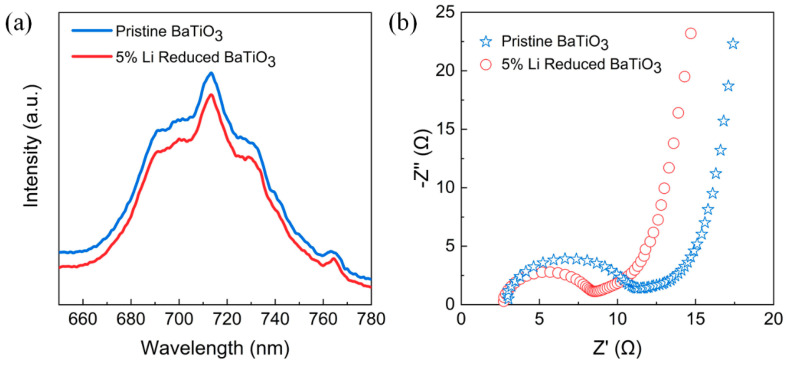
(**a**) Photoluminescence spectra and (**b**) the electrochemical impedance spectra of pure BTO and BTO-5, respectively.

Figure 7a shows the photoluminescence (PL) spectra of pristine BTO and BTO-5. Both samples exhibit an emission peak at 713 nm when excited by 325 nm light. However, the fluorescence emission intensity of BTO-5 is slightly lower compared to pristine BTO. This decrease in intensity generally suggests that BTO-5 has less recombination of excited-state electron–hole pairs, leading to a longer carrier lifetime. Additionally, electrochemical impedance spectroscopy (EIS) was performed to evaluate the efficiency of electron–hole pair migration. As presented in Figure 7b, BTO-5 displays a smaller arc radius compared to pristine BTO. This indicates that the charge carriers in BTO-5 have improved emigration and transfer efficiencies due to the implanted defects. The smaller arc radius suggests that the charge carriers can move more easily and efficiently, enhancing the piezocatalytic activity of BTO-5. The combination of PL and EIS results confirmed that the oxygen vacancies in BTO play a crucial role in promoting effective charge carrier separation and facilitating their transfer. This improved charge carrier behavior contributes to the enhanced piezocatalytic activity of BTO-5.

**Figure 8:**
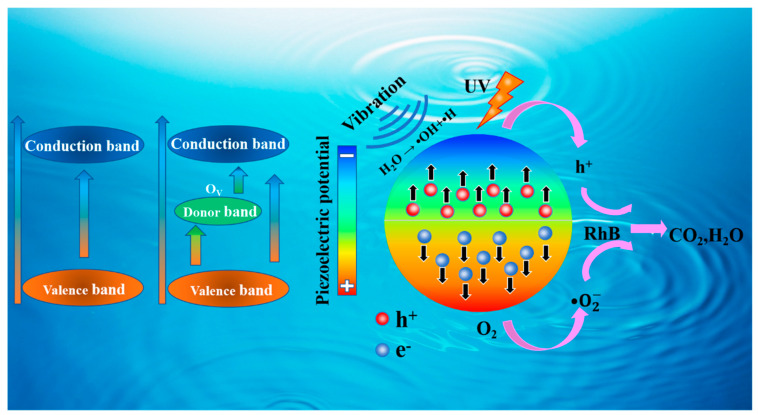
A schematic diagram of RhB degradation via the synergistically piezocatalytic effect of BaTiO_3_ with oxygen vacancies.

In Figure 8, a schematic diagram illustrates the degradation of RhB through the synergistical piezocatalytic effect of BaTiO_3_ with oxygen vacancies. The presence of free charge carriers plays a crucial role in the catalysis process. It is well known that oxygen vacancies in BTO create a shallow donor band near the conduction band, facilitating the transition of photogenerated electrons from the valence band to the conduction band. This enhances the photocatalytic performance of BTO by increasing the concentration of free carriers. Furthermore, oxygen vacancies enhance the adsorption of O_2_, which is favorable for generating more hydroxyl groups through the ultrasonic cavitation effect [26]. The increased presence of hydroxyl groups, particularly ·OH radicals, contributes to the degradation of RhB molecules. Oxygen vacancies also lead to a decrease in the elastic modulus of BTO [58]. Under the external stress generated by ultrasonic vibration, the “softer” BTO is more prone to bending, resulting in the generation of free carriers at a faster rate. In addition, BTO with oxygen vacancies exhibits higher surface conductivity, which is beneficial to the transmission of free carriers. Due to the piezoelectric polarization, a certain amount of positive and negative charges will be generated on the surface of the BTO, and an internal polarization electric field will be established. The internal polarization electric field can effectively attract the electrons and holes generated in the reaction to move in the opposite direction, thereby improving the catalytic performance of BTO.

## 5. Conclusions

In summary, BTO nanoparticles with varying concentrations of oxygen vacancy were synthesized by grinding BTO with lithium metal powders at room temperature. The concentration of oxygen vacancies was found to have a significant impact on the piezocatalytic performance of BTO nanoparticles. Remarkably, BTO with an optimized concentration of oxygen vacancies exhibited the highest reaction rate constant of 0.1721 min^−1^, which is approximately four times higher than that of pristine BTO. This also demonstrates that the synergistic coupling effect of oxygen vacancies and piezoelectric catalysis-ultrasonic cavitation can generate a considerable amount of polarization charges and induce the formation of reactive superoxide radicals (·O^2−^) and holes (h^+^), which further accelerate the charge carrier separation and promote carrier transport, resulting in the enhancement of piezocatalytic efficiency. This study not only demonstrated the influence of oxygen vacancies on the piezocatalytic performance of BTO but also provided a simple and scalable method for modulating the concentration of oxygen vacancies in piezoelectric oxide catalysts at room temperature.

## Figures and Tables

**Table 1:** Comparison of degradation efficiencies of various piezocatalysts.

Piezocatalyst	Dye Species	Vibration Source	Light Source	Initial Dye Concentration	Catalyst Dosage	Rate Constant	Ref.
BTO nanoparticles	RhB	150 W, 40 kHz	125 W, 365 nm	5 mg/L	1 g/L	0.1721 min^−1^	This work
BTO nanoparticles	RhB	150 W, 40 kHz	dark	5 mg/L	1 g/L	0.1189 min^−1^	This work
BTO nanobelts	RhB	100 W, 50 kHz	dark	10 mg/L	1 g/L	0.0253 min^−1^	[26]
BTO nanosheets	MO	100 W, 40 kHz	/	5 mg/L	1 g/L	0.1279 min^−1^	[54]
BTO nanowires	RhB	80 W,	/	5 mg/L	/	<0.016 min^−1^	[12]
BTO/TiO_2_ nanofibers	RhB	300 W, 40 kHz	250 W, 365 nm	5 mg/L	1 g/L	0.0967 min^−1^	[10]
BTO nanofibers	RhB	100 W, 40 kHz	/	7.5 mg/L	1 g/L	0.0736 min^−1^	[55]
ZnO/BTO heterostructures	RhB	120 W, 40 kHz	150 W	10 mg/L	1 g/L	0.118 min^−1^	[56]

## Data Availability

Data are contained within the article.

## Supplementary Materials

The following supporting information can be downloaded at: https://www.mdpi.com/article/10.3390/nano14030276/s1, Figure S1. SEM images of BaTiO_3_-*x* with various of Li powder treatment. (a) 0 wt%, (b) 1 wt%, (c) 3 wt%, (d) 5 wt%, (e) 7 wt%, (f) 10 wt%. The scale bar is 200 nm. Figure S2. XPS spectra (Li 1s) of lithium reduced BaTiO_3_ nanoparticles. It is apparent that no peaks correspond to Li (55.5 eV) can be found in the samples, which means the generated lithium oxides have been dissolved and removed in the washing processes. Figure S3. The effect of BaTiO_3_ treated with Li (wt%) on the degradation of RhB under UV irradiation without vabiration. Figure S4. The absorption spectroscopy of BaTiO_3_ powders treated with different Li (wt%). Their band gap has barely changed. Figure S5. (a) Cyclic stability of BTO-5. (b) XRD patterns and (c) room-temperature Raman spectra of the BTO-5 piezocatalysts before and after vibrational catalysis.
