# Supplementary material for: Boosting Piezocatalytic Performance of BaTiO3 by Tuning Defects at Room Temperature
Source: Nanomaterials (Basel). 2024 Jan 29;14(3):276. doi: 10.3390/nano14030276 (PMC10857094; doi:10.3390/nano14030276)
Supplement: Supplementary file 1 [file nanomaterials-14-00276-s001.zip › nanomaterials-2783248-supplementary.pdf]

Supplementary Information

Article

Boosting Piezocatalytic Performance of $BaTiO_3$ by Tuning Defects at Room Temperature

Donghui An [1,2], Renhong Liang [1,2], Hua Liu [1], Chao Zhou [1], Mao Ye [1], Renkui Zheng [1,2], Han Li [1,*] and Shanming Ke [1,2,*]

[1] School of Physics and Materials Science, Guangzhou University, Guangzhou 510006, PR China
[2] School of Physics and Materials Science, Nanchang University, Nanchang 330031, PR China
* Correspondence: lihan@gzhu.edu.cn (H. Li); ksm@gzhu.edu.cn (S. Ke)

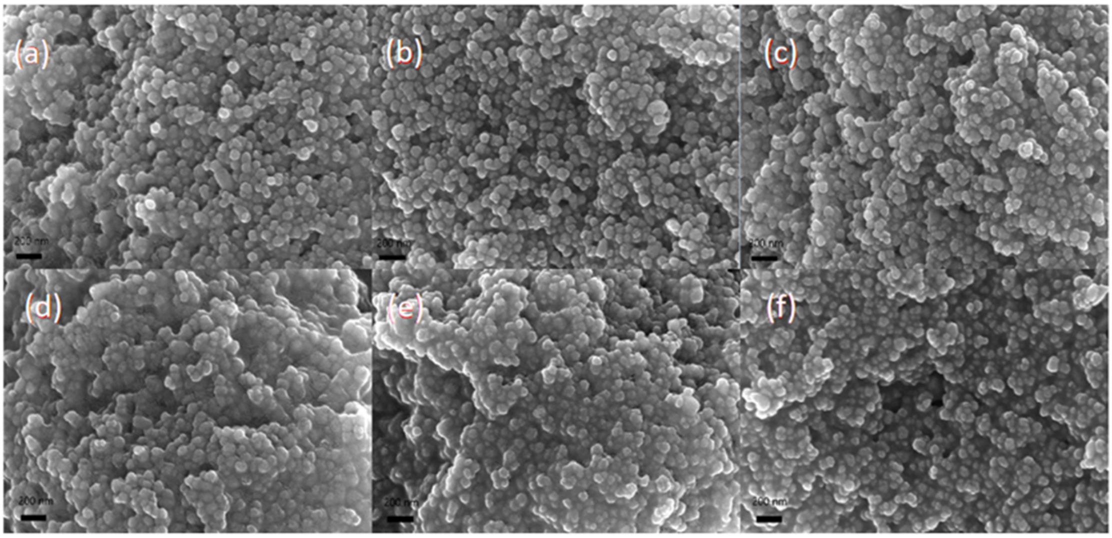

Figure S1. SEM images of $BaTiO_{3-x}$ with various of Li powder treatment. (a) 0 wt%, (b) 1 wt%, (c) 3 wt%, (d) 5 wt%, (e) 7 wt%, (f) 10 wt%. The scale bar is 200 nm.

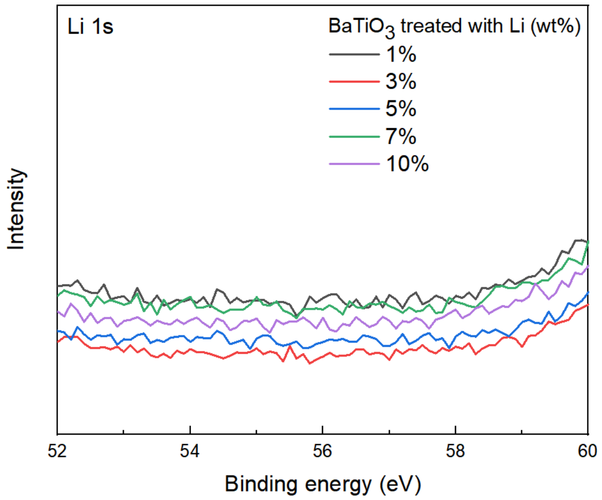

Figure S2. XPS spectra (Li 1s) of lithium reduced $BaTiO_3$ nanoparticles. It is apparent that no peaks correspond to Li (55.5 eV) can be found in the samples, which means the generated lithium oxides have been dissolved and removed in the washing processes.

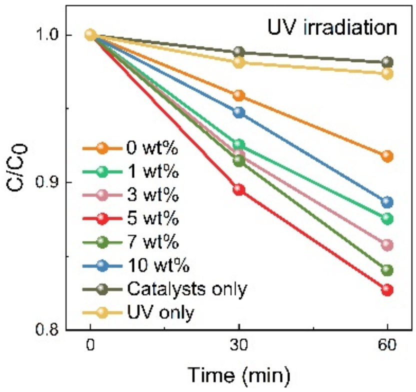

Figure S3. The effect of $BaTiO_3$ treated with Li (wt %) on the degradation of RhB under UV irradiation without vabiration.

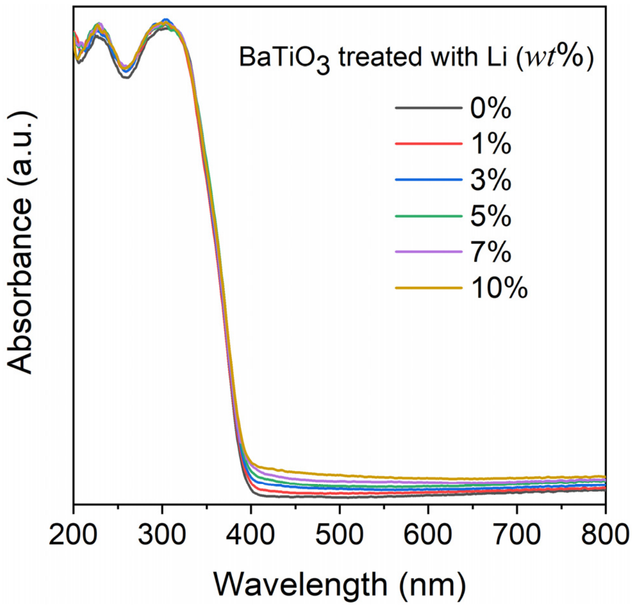

Figure S4. The absorption spectroscopy of $BaTiO_3$ powders treated with different Li (wt%). Their band gap has barely changed.

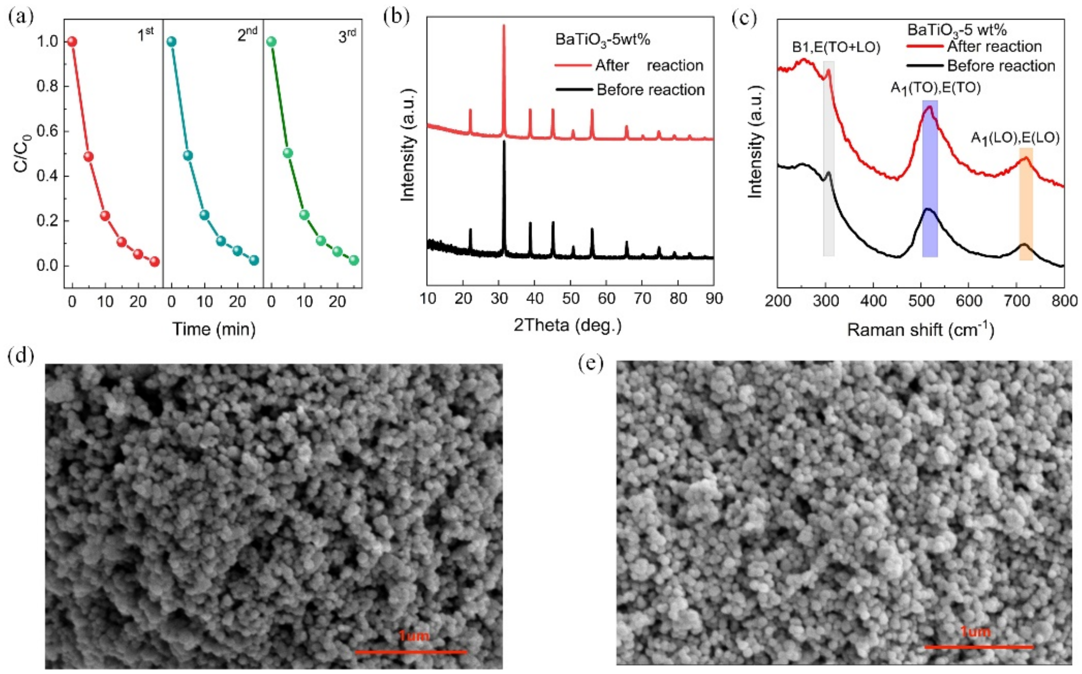

Figure S5. (a) Cyclic stability of BTO-5. (b) XRD patterns and (c) room-temperature Raman spectra of the BTO-5 piezocatalysts before and after vibrational catalysis.
